# Characterization of the complete mitogenomes of two species of Eumolpinae (Coleoptera: Chrysomelidae) and phylogenetic insights

**DOI:** 10.1093/jisesa/ieag037

**Published:** 2026-05-05

**Authors:** Hathal M Al Dhafer, Mahmoud S Abdel-Dayem, Xiao-Ling Lin, Qi Fang, Ioannis Eleftherianos, Amr Mohamed, Rui-E Nie

**Affiliations:** King Saud University Museum of Arthropods (KSMA), Plant Protection Department, College of Food and Agriculture Sciences, King Saud University, Riyadh, Saudi Arabia; King Saud University Museum of Arthropods (KSMA), Plant Protection Department, College of Food and Agriculture Sciences, King Saud University, Riyadh, Saudi Arabia; The Anhui Provincial Key Laboratory of Biodiversity Conservation and Ecological Security in the Yangtze River Basin, College of Life Sciences, Anhui Normal University, Wuhu, Anhui, China; The Anhui Provincial Key Laboratory of Biodiversity Conservation and Ecological Security in the Yangtze River Basin, College of Life Sciences, Anhui Normal University, Wuhu, Anhui, China; School of Biological Sciences, Institute for Global Food Security, Queen’s University Belfast, Belfast, UK; Department of Entomology, Faculty of Science, Cairo University, Giza, Egypt; The Anhui Provincial Key Laboratory of Biodiversity Conservation and Ecological Security in the Yangtze River Basin, College of Life Sciences, Anhui Normal University, Wuhu, Anhui, China

**Keywords:** Bromiini, Ka/Ks ratio, arid-adaptation, tRNA structure, purifying selection

## Abstract

The subfamily Eumolpinae holds substantial economic importance due to their plant-feeding habits. In this study, 2 complete mitochondrial genomes of eumolpine leaf beetles, *Macrocoma budura* Daccordi & Medvedev and *Colasposoma grande grande* (Lefèvre), were newly sequenced. Both mitogenomes possess the standard 37 mitochondrial genes and a large noncoding control region, and exhibit a highly conserved gene arrangement except for a diagnostic inversion of *trnR* (*tRNA-Arg*) and *trnA* (*tRNA-Ala*). The inversion of these 2 tRNAs has been reported previously in Eumolpinae and represents a potential subfamily-level synapomorphy. Most tRNAs adopt the typical cloverleaf conformation, with the exception of *C. g. grande trnS1* (*tRNA-Ser [AGN]*), which lacks the DHU (dihydrouridine [tRNA structural domain]) arm, and the *M. budura tRNA-Cys*, which has a shortened D-loop, suggesting possible lineage-specific structural variation. Bayesian and maximum likelihood analyses recover *C. g. grande* within Euryopini, whereas *M. budura* is placed within Bromiini with an intratribal position that varies by dataset. These new mitogenomes not only contribute to the sparse genomic resources for Eumolpinae from arid regions but also demonstrate the value of mitochondrial gene order and sequence data to resolve species-level relationships and biogeographic histories within Chrysomelidae.

## Introduction

The subfamily Eumolpinae (Coleoptera: Chrysomelidae) contains over 7,000 identified species worldwide with remarkable morphological and ecological diversity (Jolivet et al. 2014). As herbivorous beetles, Eumolpinae hold substantial economic importance due to their plant-feeding habits. Many species are agricultural pests, damaging crops such as cotton, sweet potatoes, citrus, and palms, leading to significant yield losses and necessitating pest control measures (Jolivet and Verma 2002, Fernandez and Hilker 2007, Beenen and Roques 2010, Papadopoulou et al. 2013). Additionally, their unresolved taxonomy and high species richness, especially in tropical regions, present critical opportunities for systematic and biogeographic studies. The phylogenetic researches of this subfamily based on molecular and morphological data have been studied to some extent, but the phylogenetic relationships of some groups remain unresolved (Jolivet and Verma 2008, Reid 2017, Bezdek and Sekerka 2024, Nie et al. 2024). These challenges are associated with the so-called Linnaean shortfall, where gaps in taxonomic knowledge impede advancements in other research areas (Vergara-Asenjo et al. 2023).

Mitochondrial genomes (mitogenomes) are compact (14 to 20 kb in insects), maternally inherited, and evolve at high rates, making them as valuable markers for species- and subfamily-level systematics (Bernt et al. 2013a, Cameron 2025). In Coleoptera, the most species-rich insect order, mitogenomic data have clarified relationships at several taxonomic levels (Gómez-Rodríguez et al. 2015, Yuan et al. 2016, Nie et al. 2020, 2021), but many groups remain undersampled. The features of insects’ mitogenome such as rearranged gene arrangements, noncanonical start/stop codons, and tRNA secondary-structure variants have proven phylogenetically informative in Coleoptera (Wei et al. 2010, Song et al. 2018, Feng et al. 2020, Liu et al. 2020, Nie et al. 2020, Shen et al. 2022, Yang et al. 2022, Liao et al. 2024, Huang et al. 2025, Kirsch et al. 2025). Mitochondrial tRNA cluster inversions have been proposed as potential synapomorphies for eumolpine lineage (Nie et al. 2020). However, their occurrence, evolutionary origins, and functional implications in Eumolpinae particularly among taxa inhabiting arid ecosystems such as the Arabian Peninsula remain unexplored. This region, a known hotspot for beetle endemism and diversity (Abdel-Dayem et al. 2020, 2025), presents unique climatic and ecological pressures that may have driven divergent evolutionary trajectories in Eumolpinae. Investigating mitogenomic variation in these taxa could reveal novel evolutionary patterns and provide critical insights into the subfamily’s diversification history.

The advancement of next-generation sequencing (NGS) technologies has revolutionized mitogenomic data acquisition, enabling more robust phylogenetic reconstructions across diverse insect lineages (Moreno-Carmona et al. 2021, Macken et al. 2023). However, significant gaps persist in genomic representation among Coleoptera, particularly within the Eumolpinae subfamily, which remains markedly underrepresented despite these technological advances. So far, only 11 complete mitogenomes of eumolpine species were publicly available—a limitation that inhibits robust phylogenetic inference and masks evolutionary characteristics such as gene rearrangements and codon-usage biases that may define large clades or react to biogeographic history.

Within the subfamily Eumolpinae, the tribe Bromiini is one of the largest lineages, characterized by a setaceous or scaled integument and a subcylindrical prosternum. It includes genera such as *Macrocoma*, *Pseudocolaspis*, and *Colaspidea* (Moseyko and Sprecher-Uebersax 2010, Jolivet et al. 2014). In contrast, Euryopini is a less speciose tribe within Eumolpinae, including genera such as *Colasposoma* (Seeno and Wilcox 1982, Bezdek and Sekerka 2024). In this study, we newly sequenced the complete mitogenomes of 2 Eumolpinae species from southwestern Saudi Arabia: *Macrocoma budura* (Bromiini; Daccordi & Medvedev) and *Colasposoma grande grande* (Bromiini; Lefèvre). *Macrocoma budura* (4 to 6 mm) is a Saudi endemic restricted to montane wadis (eg Al-Magardah, 411 m elevation), where adults feed on *Indigofera* spp. and other native leguminous shrubs (Fabaceae) (Bryant 1957, Warchałowski 2001). *Colasposoma g. grande* (7 to 9 mm) is a widespread Arabian species found in arid lowlands (eg Al Bahah, Saudi Arabia, 892 m). *Colasposoma g. grande* was originally described as *Eryxia grandis*; the latter was transferred to the genus *Colasposoma* by Zoia (2012). This species is associated with *Acacia* and *Ziziphus* (Rhamnaceae). It is noted for its metallic green-blue elytra (Löbl and Smetana 2010, Zoia 2012). Both species represent understudied taxa within a subfamily that includes important pests of date palms (Arecaceae)—a key regional crop.

The primary aim of this study is to provide a comprehensive analysis of the mitogenomes of *M. budura* and *C. g. grande* in order to: (i) investigate their gene organization and identify structural synapomorphies, such as tRNA inversions, (ii) assess base composition biases within these genomes, and (iii) reconstruct species-level phylogenies to refine our understanding of the evolutionary relationships within the Bromiini tribe and the broader subfamily. By comparing key features, evolutionary rates, base compositions, and relative synonymous codon usage (RSCU) across species, we aim to refine the phylogenetic framework of Eumolpinae and contribute to a deeper understanding of their molecular evolution.

## Materials and Methods

### Sample Collection and DNA Extraction

Specimens of *M. budura* and *C. g. grande* were collected from 2 locations in the Kingdom of Saudi Arabia. The *M. budura* specimen was collected by M.S. Abdel-Dayem on 2 June 2012 from Al-Magardah, W. Yabah (19°16.271′N, 41°48.464′E; elevation: 411 m). The *C. g. grande* specimen was collected by Al Dhafer et al. on 15 November 2015 from Al Bahah, Shada Al Ala Housing (19°52.598′N, 41°18.672′E; elevation: 892 m).

The collected specimens were air-dried and preserved in specimen boxes prior to DNA extraction. Genomic DNA was extracted from the head and prothorax using the DNeasy Blood and Tissue Kit (TIANGEN, Beijing, China), eluted in 100 µl of TE (tris–EDTA buffer) buffer, and stored at −80 °C until further use. Notably, the dried specimens were soaked overnight in a phosphate ion solution prior to DNA extraction, and all subsequent procedures were performed on ice to maintain sample integrity.

Voucher specimens of both taxa are deposited in the Anhui Provincial Key Laboratory of the Conservation and Exploitation of Biological Resources, College of Life Sciences, Anhui Normal University, Anhui, China. Imaging was conducted using a Nikon SMZ 1500 digital camera mounted on a stereo microscope. Final images were processed using Helicon Focus 8.1 and Adobe Photoshop 2023.

### Genome Sequencing, Mitogenome Assembly, and Annotation

Genomic DNA was used to sequence the mitochondrial genomes through high-throughput sequencing on the Illumina NovaSeq 6000 platform at Berry Genomics Corporation (Beijing, China). Libraries were prepared with 150 bp paired-end reads and an insert size of 350 bp, with 1 sequencing library generated per specimen. Adapter trimming was performed using Trimmomatic v0.36 (Bolger et al. 2014), and low-quality or short reads were filtered out using PRINSEQ (Schmieder and Edwards 2011). The remaining high-quality reads were *de novo* assembled using GetOrganelle v1.7.7.0, employing k-mer sizes of 21, 45, 65, 85, and 105, with a *t*-value of 15 (Jin et al. 2020).

Gene annotation, assessment of mitochondrial genome circularization, and extraction of individual protein-coding genes (PCGs) and rRNA genes were performed using Geneious Prime 2025.0.2 (Kearse et al. 2012). Base composition analyses were conducted in MEGA v11 (Tamura et al. 2021). AT- and GC-skews were calculated using the following formulas: AT-skew = (A% − T%)/(A% + T%) and GC-skew = (G% − C%)/(G% + C%) (Perna and Kocher 1995). Codon usage and RSCU for the 13 PCGs were calculated using PhyloSuite (Zhang et al. 2020, Xiang et al. 2023). Mitogenome maps were generated using the CGView Server (http://cgview.ca, accessed 24 March 2025) (Grant and Stothard 2008).

The secondary structures and anticodons of mitochondrial tRNAs were predicted using the MITOS (MITOchondrial Genome Annotation Server) Web Server (http://mitos2.bioinf.uni-leipzig.de/index.py, accessed 24 March 2025) (Bernt et al. 2013b) and tRNAscan-SE 2.0 (http://trna.ucsc.edu/tRNAscan-SE, accessed 24 March 2025) (Lowe and Chan 2016). The nonsynonymous (Ka) to synonymous (Ks) substitution rates (Ka/Ks) of the 13 PCGs were calculated pairwise among 21 species of the subfamily Eumolpinae using DnaSP v6.0 (Rozas et al. 2017). Before constructing the phylogenetic tree, substitution saturation analysis of the first, second, and third codon positions of the 13 PCGs (13PCGs_codon1, 13PCGs_codon2, 13PCGs_codon3) was conducted in DAMBE v7 under the GTR model (Xia 2018).

### Phylogenetic Analyses

The phylogenetic positions of *M. budura* and *C. g. grande* were inferred based on mitochondrial genome sequences from 21 species within the subfamily Eumolpinae, with 7 species from the subfamily Cryptocephalinae used as outgroups (Table 1). Phylogenetic analyses were conducted using 2 datasets: (i) 15 genes (13PCGs + 2 rRNAs), consisting of all codons from the 13 PCGs and 2 ribosomal RNA (rRNA) genes, and (ii) 13PCGs_AA, in which the 13 PCGs were translated into amino acid sequences.

**Table 1.** List of reference mitochondrial genomes chosen for phylogenetic analysis (alphabetical by species)

Subfamily	Tribe	Species	Length (bp)	Acc. No.	References
**Eumolpinae**	Bromiini	*Aoria cyanea*	8,769	QINL006	Nie et al. 2024
**Eumolpinae**	Bromiini	*Aoria* sp.	8,225	QINL004	Nie et al. 2024
**Eumolpinae**	Typophorini	*Basilepta fulvipes*	15,762	NC_054189	Liu et al. 2020
**Eumolpinae**	Typophorini	*Basilepta melanopus*	15,905	NC_066974	Unpublished
**Eumolpinae**	Typophorini	*Basilepta* sp.	15,997	QINL131	Nie et al. 2024
**Eumolpinae**	Bromiini	*Bromius obscurus*	16,285	KX087249	Unpublished
**Eumolpinae**	Eumolpini	*Chalcophana* sp.	15,639	MK049856	Unpublished
**Eumolpinae**	Eumolpini	*Chrysochus chinensis*	15,694	MK049871	Nie et al. 2024
**Eumolpinae**	Eumolpini	*Chrysodinopsis* sp.	15,903	KY039111	Song et al. 2018
**Eumolpinae**	Typophorini	*Cleoporus variabilis*	14,980	QINL049	Nie et al. 2024
**Eumolpinae**	Bromiini	*Colaspidea globosa*	6,481	KX943381	Gómez-Rodríguez et al. 2015
**Eumolpinae**	Euryopini	*Colasposoma dauricum*	15,490	MW354515	Unpublished
**Eumolpinae**	Euryopini	*Colasposoma grande grande*	15,565	PV591962	This study
**Cryptocephalinae**	Cryptocephalini	*Cryptocephalus ramburii*	15,789	KX943509	Gómez-Rodríguez et al. 2015
**Cryptocephalinae**	Clytrini	*Clytra espanoli*	16,005	KX943426	Gómez-Rodríguez et al. 2015
**Cryptocephalinae**	Clytrini	*Clytra quadripunctata*	15,948	KX943461	Gómez-Rodríguez et al. 2015
**Eumolpinae**	Bromiini	*Demotina* sp.1	14,897	QINL022	Nie et al. 2024
**Eumolpinae**	Bromiini	*Demotina* sp.2	15,900	QINL086	Nie et al. 2024
**Eumolpinae**	Bromiini	*Demotina* sp.3	17,215	QINL128	Nie et al. 2024
**Cryptocephalinae**	Cryptocephalini	*Labidostomis taxicornis*	15,783	KX943474	Gómez-Rodríguez et al. 2015
**Eumolpinae**	Bromiini	*Macrocoma budura*	15,374	PV591961	This study
**Cryptocephalinae**	Pachybrachini	*Pachybrachis suffrianii*	15,760	KX943489	Nie et al. 2020
**Eumolpinae**	Eumolpini	*Platycorynus* sp.	16,130	MK049872	Nie et al. 2020
**Cryptocephalinae**	Cryptocephalini	*Physosmaragdina nigrifrons*	15,902	MT554389	Unpublished
**Eumolpinae**	Bromiini^a^	*Pseudocolaspis* sp.	16,287	JX412756	Unpublished
**Cryptocephalinae**	Cryptocephalini	*Stylosomus ilicicola*	15,758	KX943463	Gómez-Rodríguez et al. 2015
**Eumolpinae**	Synetinae^b^	*Syneta adamsi*	15,511	MK049876	Nie et al. 2020
**Eumolpinae**	Typophorini	*Trichochrysea japana*	15,681	OR387477	Unpublished

Tribal assignments follow current tribal-level classifications of Chrysomelidae, based on widely used taxonomic frameworks (eg Bezdek and Sekerka 2024) and recent phylogenetic studies.

a *Pseudocolaspis* may fall under Bromiini or Eumolpini depending on classification; genome studies often place it with bromine-like clades. However, we placed it under Bromiini as per Cardoso and Gómez-Zurita (2025).

b *Syneta* is sometimes treated in its own subfamily (Synetinae) separate from traditional Eumolpinae.

The 13 PCGs and 2 rRNAs were extracted from annotated mitochondrial genomes using Geneious Prime 2025.0.2. Each gene was aligned separately using MUSCLE in MEGA 64 with default parameters (Edgar 2004). The resulting alignments were imported into PhyloSuite v1.2.3 (Zhang et al. 2020, Xiang et al. 2023), where trimming and concatenation were performed. Poorly aligned regions and ambiguous sites were removed using Gblocks for nucleotide sequences (Castresana 2000) and trimAl for amino acid and rRNA sequences (Capella-Gutiérrez et al. 2009). The final concatenated alignments were generated using the “Concatenate Sequence” function in PhyloSuite.

Bayesian inference (BI) analyses were conducted for both datasets to reconstruct the phylogenetic relationships within Eumolpinae. Phylogenetic trees were inferred using PhyloBayes MPI v1.5a under the CAT-GTR model (Lartillot et al. 2013). Two independent, parallel Markov chain Monte Carlo runs were performed for each dataset until the maximum discrepancy (maxdiff) between the chains was less than 0.1. A consensus tree was generated from the remaining trees after discarding the initial 25% of trees as burn-in. Maximum likelihood (ML) analysis was conducted in IQ-TREE v2.4.0 (Minh et al. 2020) with the best-fit model selected automatically (MFP) and node support assessed with 1,000 SH-aLRT replicates (Guindon et al. 2010) and 1,000 UFBoot2 bootstraps (Hoang et al. 2018). The ML topology (shown in the main text) was selected for primary presentation because it is most congruent with the updated tribal assignments (Bezdek and Sekerka 2024); alternative phylogenetic reconstructions derived from Bayesian and ML analyses are provided as Supplementary Figs S3–S10 to document topological variation across analytical approaches.

### Nomenclature and Gene Annotation

In this study, we adopt the compact mitochondrial gene nomenclature commonly used in insect mitogenomics. On first mention we give both the compact symbol and the full name; thereafter we use the compact forms; specifically: *trnL1* (*tRNA-Leu [UUR]*), *trnL2* (*tRNA-Leu [CUN]*), *trnS1* (*tRNA-Ser [AGN]*), *trnS2* (*tRNA-Ser [UCN]*), *trnV* (*tRNA-Val*), *trnR* (*tRNA-Arg*), *trnA* (*tRNA-Ala*). Ribosomal RNAs are abbreviated *rrnL* (*16S rRNA*) and *rrnS* (*12S rRNA*). The noncoding AT-rich region is referred to as the control region (AT-rich region) on first mention and as control region thereafter.

## Results

### The Structure of the Mitochondrial Genomes

Raw reads (about 15 Gb) were obtained for each sample using high-throughput sequencing technology. In this study, 2 newly sequenced complete mitochondrial genomes of Eumolpinae were obtained: *M. budura* and *C. g. grande*. The mitogenome lengths of *M. budura* and *C. g. grande* are 15,374 and 15,565 bp, respectively. The primary variation in genome size between the 2 species is attributed to differences in the length of the control region.

**Fig. 1.**
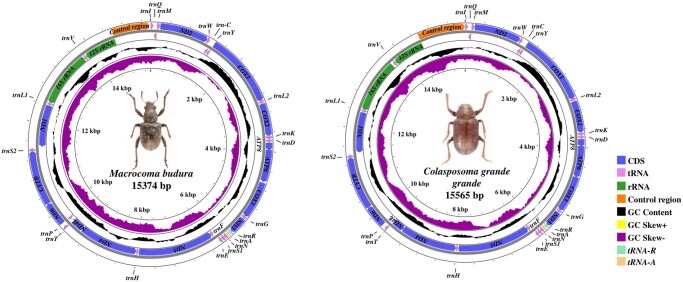
Circular maps of the complete mitochondrial genomes of *Macrocoma budura* (15,374 bp, left) and *Colasposoma grande grande* (15,565 bp, right). Genes on the outer circle are encoded on the J-strand; genes on the inner circle are encoded on the N-strand. PCGs are shown by their standard uppercase symbols (eg *COI*, *ND5*). Transfer RNAs are indicated in compact form (eg *trnR* (*tRNA*-*Arg*), *trnA* (*tRNA-Ala*), *trnV* (*tRNA-Val*)). The conserved inversion of *trnA* and *trnR* is highlighted in light orange and light green, respectively. The control region is indicated at the upper left.

Both of these mitogenomes contain 37 genes (13 PCGs, 2 rRNAs and 22 tRNAs) and 1 large noncoding region (control region) (Fig. 1). Among these 37 genes, 9 PCGs and 14 *tRNAs* were encoded on the positive strand (J-strand), while the remaining 4 PCGs, 8 *tRNAs*, and 2 *rRNAs* were encoded on the negative strand (N-strand) (Fig. 1). The organization of the 2 new mitogenomes was very compact, but there is also a certain length of overlaps and gaps (Supplementary Table S1).

However, unlike the mitogenome of most insects, the gene order of the newly obtained Eumolpinae species does not follow the presumed ancestral arrangement of the insect mitogenome. Their characteristic is that the order of the *trnR* (*tRNA-Arg*) and *trnA* (*tRNA-Ala*) genes is reversed, which is the same as the study by Nie et al. in 2020 (Fig. 1). The skew analysis indicated that the obvious bias prefers to use C rather than G. In nucleotide composition, both of the 2 new mitogenomes were significantly biased toward A and T: 77.3% in *M. budura*; 76.4% in *C. g. grande* (Supplementary Table S2). The skew metrics of 2 species showed that GC-skew was almost negative in PCGs, *tRNAs*, *rRNAs*, and the control region (Fig. 1).

### Protein-Coding Genes

There was no significant difference in the lengths of the 13 PCGs between *M. budura* and *C. g. grande*, which were 14,567 and 14,541 bp, respectively. The contents of AT were 77.4% and 75.3%, respectively (Supplementary Table S2). Among the 13 PCGs of *M. budura*, 10 genes start with the typical mitogenome codon ATN (N for C, G, and T), while *ND1* gene starts with TTG and *ND3*, *ND5* gene start with ATA (Supplementary Table S1). Among the 13 PCGs of *C. g. grande*, 8 genes start with the typical ATN, while the *ND1* gene was the same as that of *M. budura*, starting with TTG, and the *ATP8*, *ND2*, *ND4* and *ND5* genes started with ATA (Supplementary Table S1). All the stop codons of the 13 PCGs of the 2 species were TAA/TAG or incomplete stop codons by T (Supplementary Table S1).

**Fig. 2.**
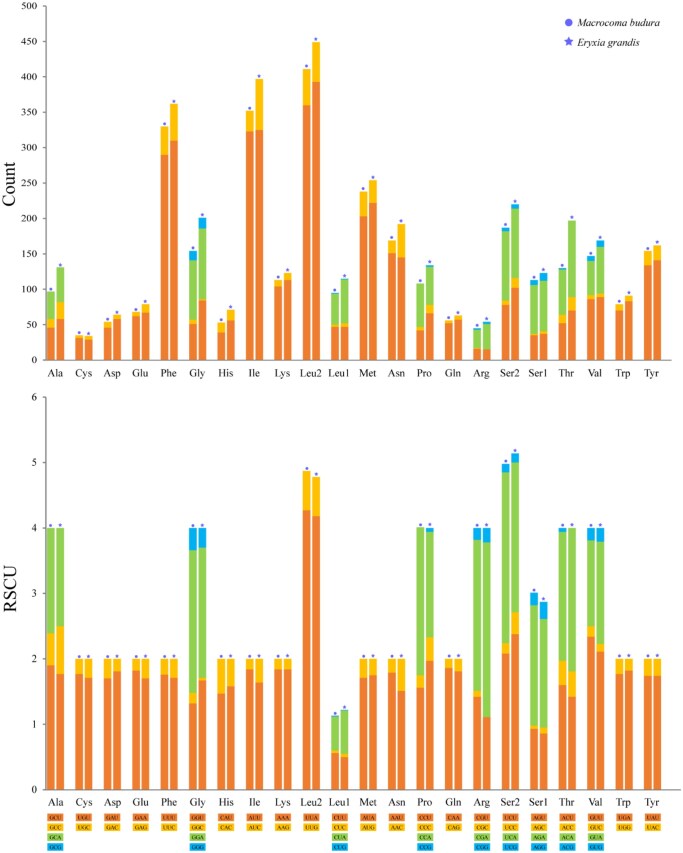
Usage of amino acids and RSCU of PCGs in the mitochondrial genome of *Macrocoma budura* and *Colasposoma grande grande*.

The usage of codons and amino acids also reflects the AT bias of the mitochondrial genome. The most frequently used amino acids in the 2 newly sequenced species were Leu2, Ile, Phe, and Met. The codons with high usage frequency (RSCU > 2.0) in *M. budura* are UUA>UCA>GUU>CGA>CCA>GGA>UCU in sequence. The codons with high usage frequency (RSCU > 2.0) in *C. g. grande* are UUA>CGA>UCU>UCA>ACA>GUU in sequence, as shown in Fig. 2.

### tRNAs, rRNAs, and Control Region

The *16S rRNA* (*rrnL*) and *12S rRNA* (*rrnS*) in both mitogenomes are located between *trnL1* (*tRNA-Leu [UUR]*) and *trnV* (*tRNA-Val*), and between *trnV* and the control region, respectively. Hereafter, we consistently use the trnX/rrnX nomenclature (eg *trnL1*, *trnV*, *rrnL*). This is similar to the mitogenome of beetles studied previously. Additionally, the *tRNA* structures of the 2 newly sequenced species are very conserved. The secondary structures of all *tRNA* are folded into the typical cloverleaf structure, except *trnS1* (*tRNA-Ser [AGN]*) of *C. g. grande*, which is characterized by the lack of DHU (dihydrouridine [tRNA structural domain])-stem and several unmatched base pairs in the anticodon stem, similar to many insects (Supplementary Figs S1 and S2). Moreover, *trnS1* (*tRNA-Ser [AGN]*) of *M. budura* has a smaller D-loop motif, and *tRNA-Cys* has a truncated D-loop motif. The lengths of the control regions of *M. budura* and *C. g. grande* were 781 and 1,017 bp, respectively, and they were located between 12S *rRNA* (*rrnS*) and *tRNA-Ile*.

**Fig. 3.**
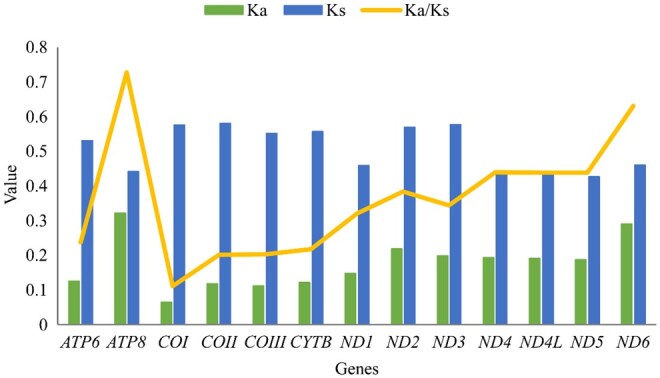
Nonsynonymous (Ka) to synonymous (Ks) substitution rates of 13 PCGs among eumolpine species.

In this study, the average ratio of nonsynonymous (Ka) to synonymous (Ks) substitution of 13 PCGs in 21 Eumolpinae was calculated, and the results are shown in Fig. 3. The Ka/Ks substitution ratios of 13 PCGs were less than 1, and ranged from 0.11170 (*COI*) to 0.72744 (*ATP8*). The results demonstrated that all PCGs were under purifying selection. The evolution rate of 13 PCGs was as follows: *ATP8 *>* ND6 *>* ND4 *>* ND4L* > *ND5 *>* ND2 *>* ND3 *>* ND1 *>* ATP6 *>* CYTB* > *COIII* > *COII* > *COI*. Among them, *COI* showed the lowest evolution rate, while *ATP8* and *ND6* exhibited a faster evolutionary rate and greater diversity than other *PCG*.

**Fig. 4.**
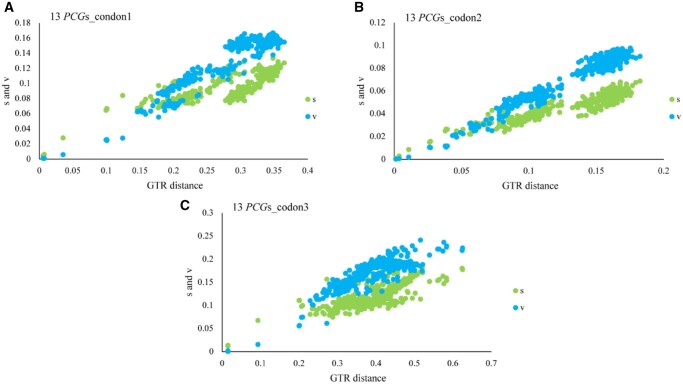
Substitution saturation plots for the 3 different mitochondrial genome datasets, showing Codon positions 1 (A), 2 (B) and 3 (C) of 13 PCGs. Each panel plot shows uncorrected pairwise divergences in transitions (s, green) and transversions (v, blue) against GTR corrected genetic distance.

Evaluation of substitution saturation indicated that all 3 codon datasets retained sufficient phylogenetic information. All the analyzed results showed a lower ISS value (index of substitution saturation) than ISS.c value (critical ISS value) (*P* < 0.05), which indicated all 3 datasets were not saturated and are feasible to use in phylogenetic analyses. In addition, substitution saturation plots further supported this conclusion (Fig. 4). Codon1 and codon2 showed a strong linear relationship between GTR distances and uncorrected pairwise divergences for both transitions and transversions, with transitions (s) typically higher than transversions (v). In contrast, codon3 exhibited a weaker linear trend and a noticeable plateau in transitions at higher divergence levels, accompanied by transversions exceeding transitions—patterns indicative of mild saturation. Nonetheless, the combination of statistical and visual evidence suggests that all 3 codon positions retain sufficient phylogenetic information.

### Phylogenetic Relationship

**Fig. 5.**
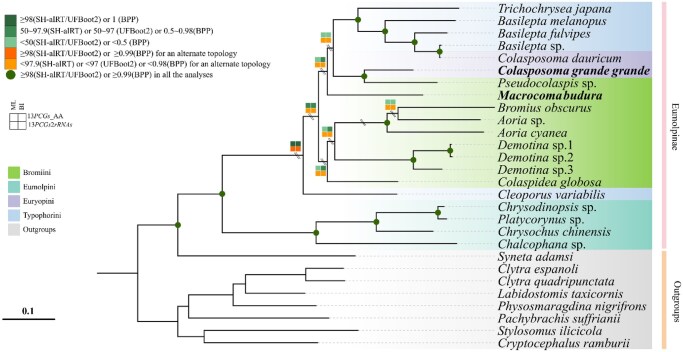
Phylogeny of Eumolpinae, inferred from ML analysis of 13 PCGs translated into amino acid sequences (13PCGs_AA). Node support values (colored squares) correspond to the ML analysis; alternative ML/Bayesian topologies and support values are provided in Supplementary Figs S3–S6. Only the lowest node support values are shown where different datasets or models yield conflicting results. Conflicting topological positions between trees are denoted by double slashes (\). The newly sequenced species in this study are marked in bold. Background colors denote different tribes (left).

To determine the phylogenetic position of *M. budura* and *C. g. grande*, we constructed phylogenetic trees based on the mitogenomes of 28 species (21 Eumolpinae as the ingroup and 7 species of Cryptocephalinae as the outgroups), using both BI and ML methods for 2 datasets: 13 PCGs + 2 rRNAs and 13 PCGs_AA (the 13 PCGs translated to amino acids). The topology of the 4 phylogenetic trees is largely congruent. For the 13 PCGs_AA dataset, both Bayesian and ML analyses recovered identical ingroup topologies. All analyses consistently recovered *C. g. grande* as sister to *Pseudocolaspis* sp., with high support in the 13PCGs_AA analyses (Fig. 5; Supplementary Figs S3–S6). In contrast, the phylogenetic position of *M. budura* varied between datasets. In both analyses of the 13 PCGs + 2 rRNAs dataset, *M. budura* clustered with *C. g. grande* and *Pseudocolaspis* sp. with moderate to high support (BPP = 0.83; SH-aLRT = 98.2; UFBoot2 = 66), the UFBoot2 = 66 indicates only moderate bootstrap support for that grouping. By contrast, in the 13 PCGs_AA dataset analyses, *M. budura* occupies a more basal position, diverging prior to the split of the *C. g. grande* and *Pseudocolaspis* sp. clade from the remaining taxa (Fig. 5). In the 13PCGs_AA ML tree *M*. *budura* branches adjacent to the *C*. *g*. *grande* + *Pseudocolaspis* clade, but its exact intratribal position is matrix- and analysis-dependent (see conflicting placements marked in Fig. 5). This ambiguity prevents strong taxonomic conclusions about *M*. *budura’*s tribal assignment from mitochondrial data alone. The *C*. *g*. *grande* + *Pseudocolaspis* clade is recovered together in our trees (Fig. 5), but because *Pseudocolaspis* is currently placed in Bromiini (Cardoso and Gómez-Zurita 2025), this topology represents a tribe-level discordance that cannot be dismissed—it may reflect taxon misplacement, mitochondrial introgression, or limited sampling; resolving whether this implies tribal reassignment requires broader taxon sampling and nuclear data. Phylogenetic analyses robustly recover *C. g. grande* as sister to *Pseudocolaspis* sp. (PP ≈ 1; SH-aLRT/UFBoot2 = 100/100 in amino-acid analyses), despite their current placement in different tribes (Euryopini and Bromiini, respectively). This result highlights a discordance between mitogenomic signal and morphology-based tribal classifications, without implying formal taxonomic reassignment. Furthermore, Eumolpini and Typophorini each form strongly supported monophyletic clades (BPP = 1; SH-aLRT/UFBoot2 = 100/100), with the notable exception of *Cleoporus variabilis*, which is placed anomalously with respect to typical Typophorini membership in our trees. These results provide new evidence for re-evaluating relationships in Bromiini and Euryopini, emphasizing the need for integrated morphological and molecular data in future Eumolpinae systematics.

## Discussion

The complete mitochondrial genomes of *M. budura* and *C. g. grande* illuminate both the conserved and distinctive elements that have been influencing eumolpine evolution. Both mitogenomes share the Coleoptera hallmarks—high A+T content (76.4% and 77.3%, respectively; Fig. 1) and the complement of all 37 genes—but also feature a characteristic inversion of *trnR* (*tRNA-Arg*) and *trnA* (*tRNA-Ala*) also first reported in other members of Eumolpinae (Nie et al. 2020). The shared inversion of *trnR* and *trnA* in our 2 newly sequenced mitogenomes (and reports from other Eumolpinae) supports the hypothesis that this rearrangement is a stable character within the subfamily and is a useful candidate synapomorphy; nevertheless, broad taxon sampling is required to test its universality and to exclude homoplastic occurrences.

Some PCGs in both taxa begin with nonstandard codons (*ND1* with TTG; *ND3*, *ND4*, *ND5*, *ATP8* with ATA) and usually terminate in 1 residue of thymine, as predicted under posttranscriptional polyadenylation (Ojala et al. 1981, Wang and Tang 2018), pointing to the evolutionarily conserved process of mitochondrial gene expression. These deviations from the ancestral insect mitogenome—though noted elsewhere within Coleoptera—may be the result of lineage-specific selective pressures or stochastic drift (Snead and Alda 2022, Nie et al. 2024, Carpenter and Vogler 2025).

Secondary structure predictions show that all tRNAs exhibit the typical cloverleaf configuration, except for *trnS1* (*tRNA-Ser [AGN]*) in *C. g. grande*, which lacks the DHU arm, and *tRNA-Cys* in *M. budura*, which has a truncated D-loop. Such structural plasticity, reflected in other beetle mitogenomes (Wei et al. 2010), is likely due to compensatory mutations preserving function under relaxed constraints. The negative GC-skew seen in coding and noncoding regions also reflects replication-strand asymmetry characteristic of AT-rich mitogenomes (Reyes et al. 1998), an influence that may subtly bias codon usage and substitution rates.

The mitochondrial phylogeny robustly recovers *C*. *g*. *grande* as sister to *Pseudocolaspis*; however, because this result is discordant with current tribal classification, its broader tribal implications should be regarded as tentative pending broader taxon sampling and nuclear data. By contrast, the position of *M*. *budura* within Bromiini is dataset-dependent (nucleotide versus amino-acid matrices) and therefore less firmly resolved. Overall, the trees improve resolution of some intratribal relationships but do not yet supersede morphology-based classifications without further corroboration (Cameron 2025). Whereas earlier authors (Warchałowski 2001, Zoia 2012) recognized broad Bromiini affinities, our mitogenomic analyses recover a strongly supported sister relationship between *C. g. grande* and *Pseudocolaspis* sp. (posterior probability ≈ 1; SH-aLRT/UFBoot2 = 100/100 in the AA dataset) (Gómez-Zurita et al. 2005), while support for *M. budura*’s inclusion in that same clade is inconsistent across matrices. Given the current taxon sampling, the strong molecular sistership between *Colasposoma* and *Pseudocolaspis* is consistent with a relatively recent common ancestry for those 2 lineages, but morphological synapomorphies should be proposed only after targeted comparative study of prosternal and elytral characters (Daccordi 1994)—we therefore frame this as a testable hypothesis rather than a demonstrated fact. The topology recovered in 1 matrix suggests that *Macrocoma* may be closely associated with the *Colasposoma + Pseudocolaspis* clade, but this relationship is not consistently recovered across datasets; therefore, any biogeographic interpretation (eg an Arabian/African xeric radiation) is preliminary and should be treated as speculative until broader sampling and nuclear data confirm the pattern. Classical treatments (Jolivet and Verma 2008) placed these genera in varying configurations; our results highlight points of concordance and conflict with morphology-based classifications, but character-level reexamination (not broad summary statements) is required to reconcile these differences. Our results (i) provide mitogenomic support for coherent tribal groupings as recovered in Fig. 5, (ii) resolve some intratribal relationships with high support (notably *Colasposoma* within Euryopini), and (iii) suggest hypotheses of regional diversification that require wider taxon and nuclear-genome sampling before proposing formal taxonomic revision or biogeographic scenarios.

Despite these advances, 2 limitations must be mentioned. First, sole reliance on mitochondrial markers can be confounded by incomplete lineage sorting or introgression (Ballard and Whitlock 2004). Integration of nuclear genomic information and broader taxon sampling—most importantly from poorly represented Eumolpinae clades—will be necessary to validate and extend our findings. Second, functional assays examining how tRNA-structure variants (eg DHU arm loss) influence mitochondrial translation efficiency could give insights into the adaptive significance of such anomalies.

In conclusion, the mitochondrial genomes of *M. budura* and *C. g. grande* provide valuable insights into the mitochondrial evolution of Eumolpinae. The inversion of *trnR* (*tRNA-Arg*) and *trnA* (*tRNA-Ala*) in these mitogenomes further supports its potential as a subfamily-level character, but we frame this as supporting evidence rather than definitive confirmation: Assessing its consistency across Eumolpinae will require expanded sampling. The structural variations in *trnS1* (*tRNA-Ser [AGN]*) and tRNA-Cys, including the loss of the DHU arm and a truncated D-loop, suggest functional plasticity within mitochondrial tRNAs, likely due to adaptive or relaxed selective pressures. Both mitogenomes exhibit a typical coleopteran mitochondrial architecture, with high AT content and negative GC-skew. Phylogenetic analysis robustly supports a close relationship between *C*. *g*. *grande* and *Pseudocolaspis* sp.; by contrast, *M*. *budura*’s placement is matrix-dependent and ambiguous, so any affiliation with Bromiini must be treated as provisional pending nuclear data and expanded sampling. Biogeographic hypotheses (eg xeric-driven diversification) are plausible but remain speculative until confirmed by denser geographic and phylogenomic sampling. Together, these findings generate testable hypotheses about tribe-level relationships in Bromiini and Eumolpinae, but they do not alone justify taxonomic revision; broader taxon sampling and nuclear data are required before proposing formal changes.

## Supplementary Material

ieag037_Supplementary_Data
