# Supplementary material for: Characterization of the complete mitogenomes of two species of Eumolpinae (Coleoptera: Chrysomelidae) and phylogenetic insights
Source: J Insect Sci. 2026 May 5;26(3):ieag037. doi: 10.1093/jisesa/ieag037 (PMC13143426; doi:10.1093/jisesa/ieag037)
Supplement: ieag037_Supplementary_Data [file ieag037_supplementary_data.pdf]

Supplementary Material

Characterization of the complete mitogenomes of two species of Eumolpinae (Coleoptera: Chrysomelidae) and insights into their phylogenies

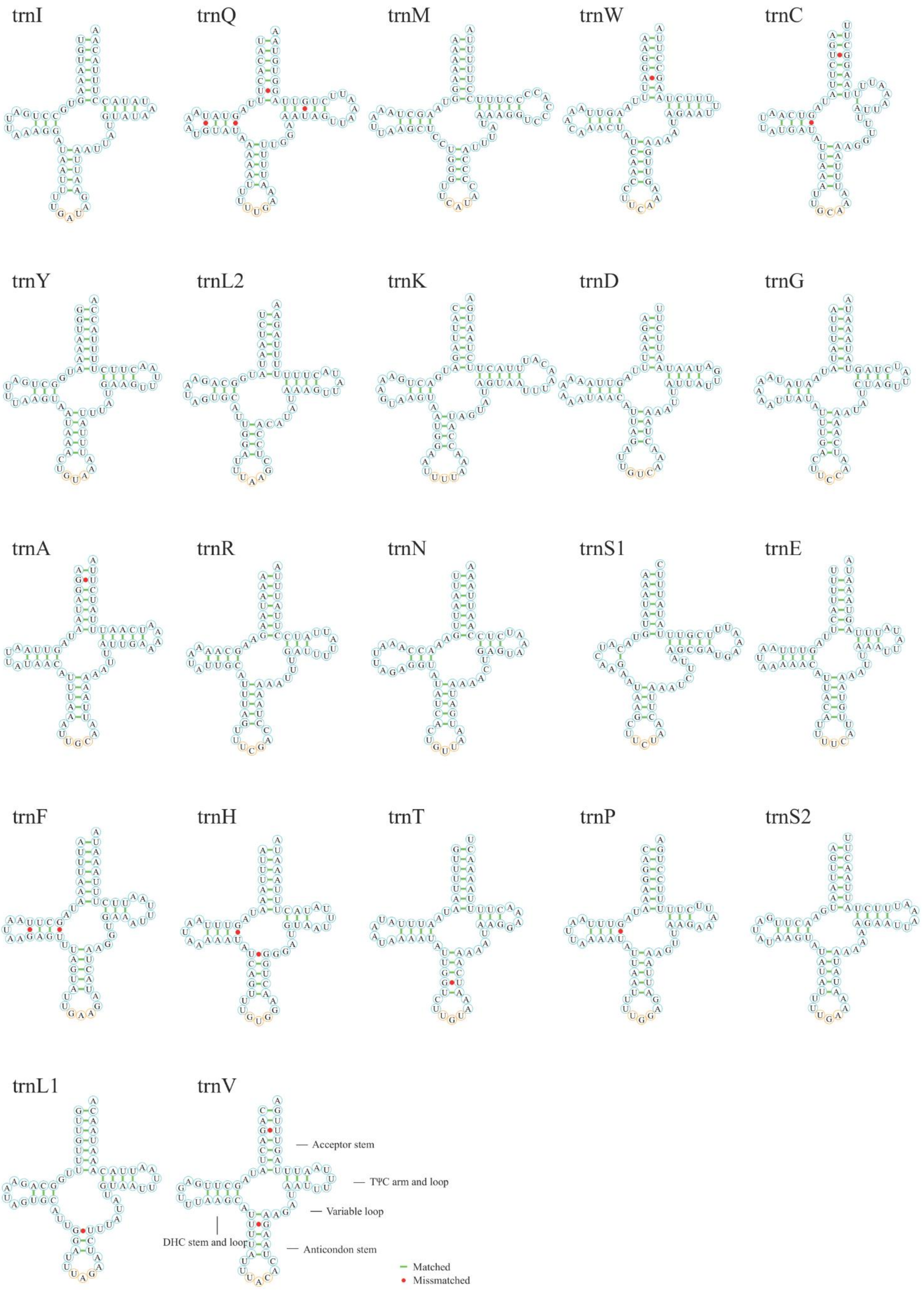

Fig. S1. Predicted secondary structures of mitochondrial tRNAs in *Macrocoma budura*.

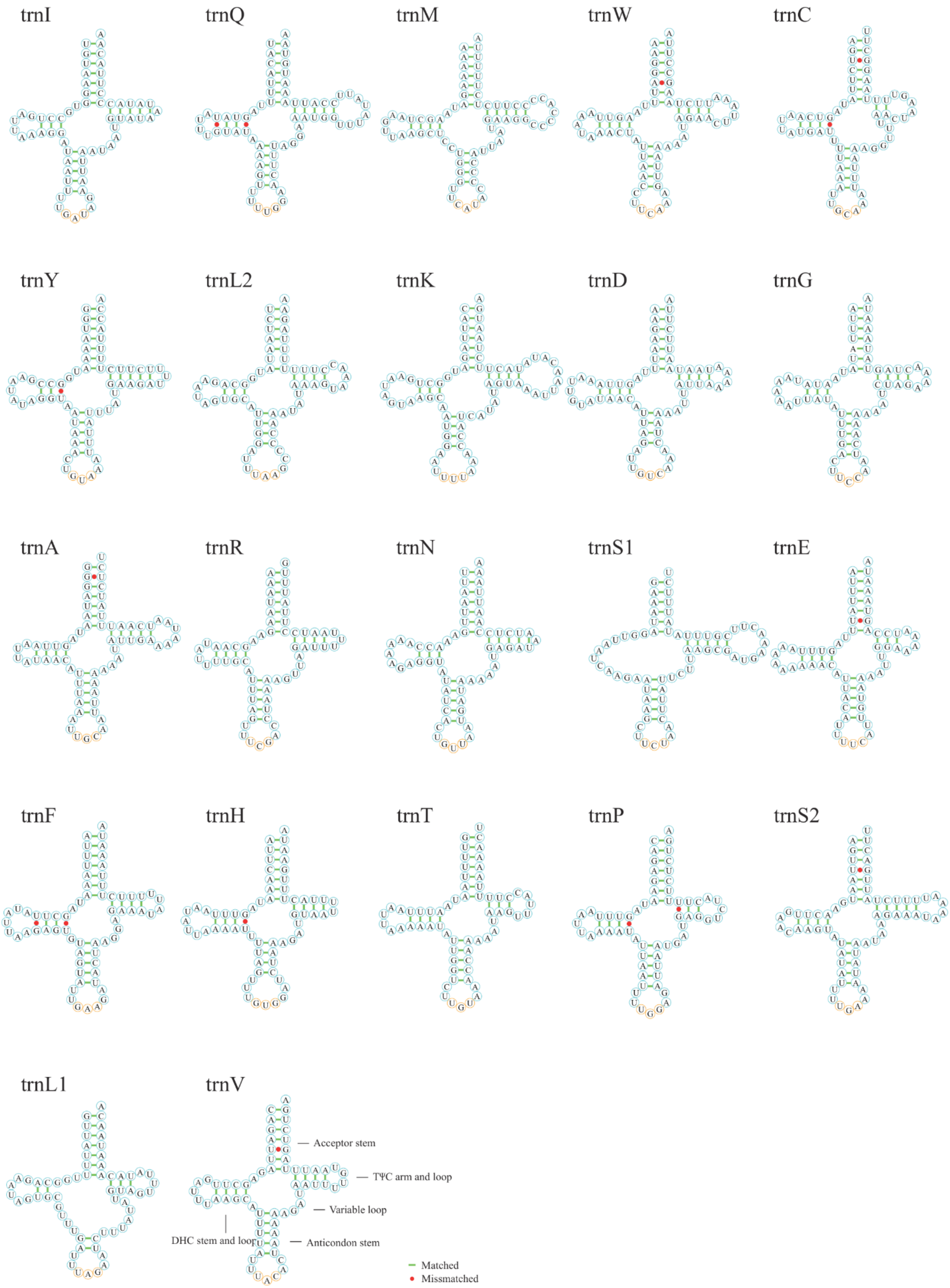

Fig. S2. Predicted secondary structures of mitochondrial tRNAs in *Colasposoma grande grande*.

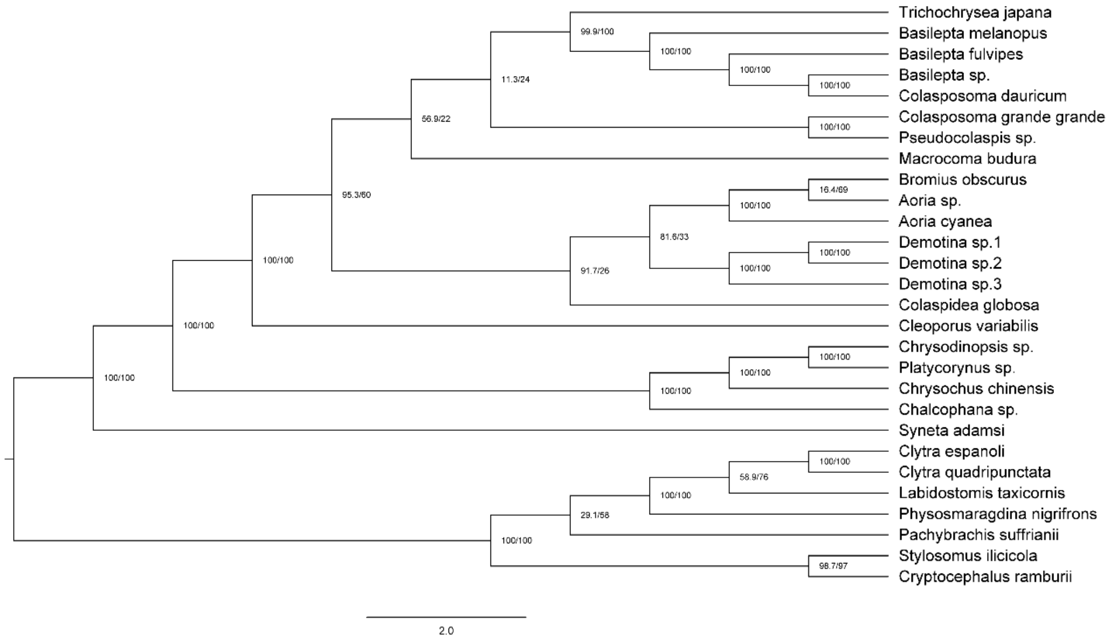

Fig. S3. Phylogenetic tree of Eumolpinae, inferred from maximum likelihood analysis (using IQ-TREE) based on 13 PCGs were translated into amino acid (13PCGs_AA). SH-aLRT and bootstrap support values are shown near the nodes.

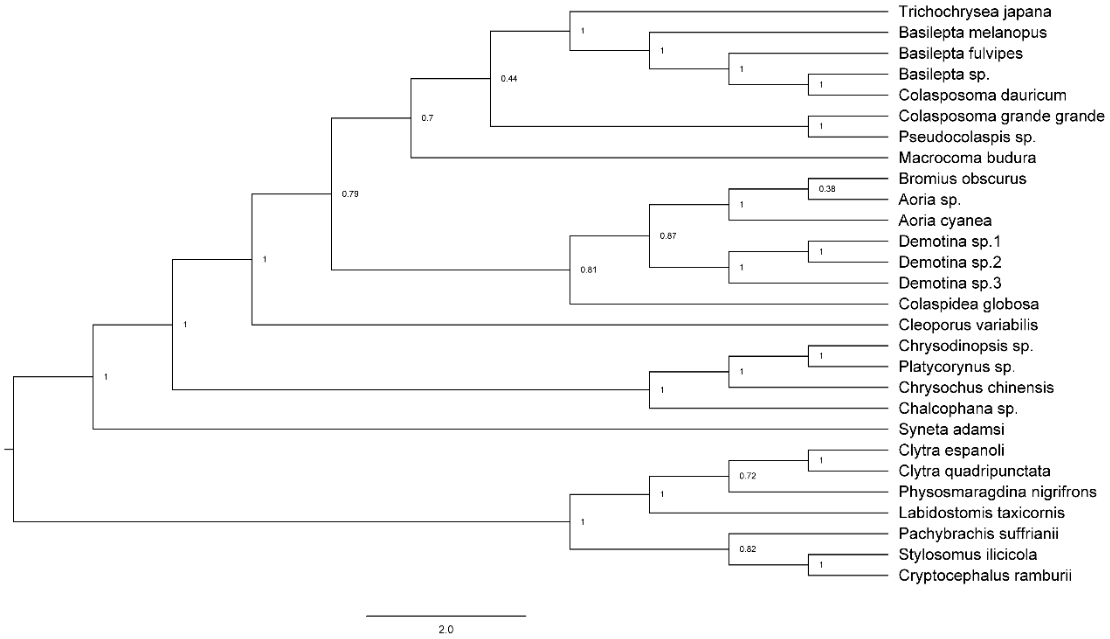

Fig. S4. Phylogenetic tree of Eumolpinae, inferred from bayesian inference (using PhyloBayes) based on 13 PCGs were translated into amino acid (13PCGs_AA). SH-aLRT and bootstrap support values are shown near the nodes.

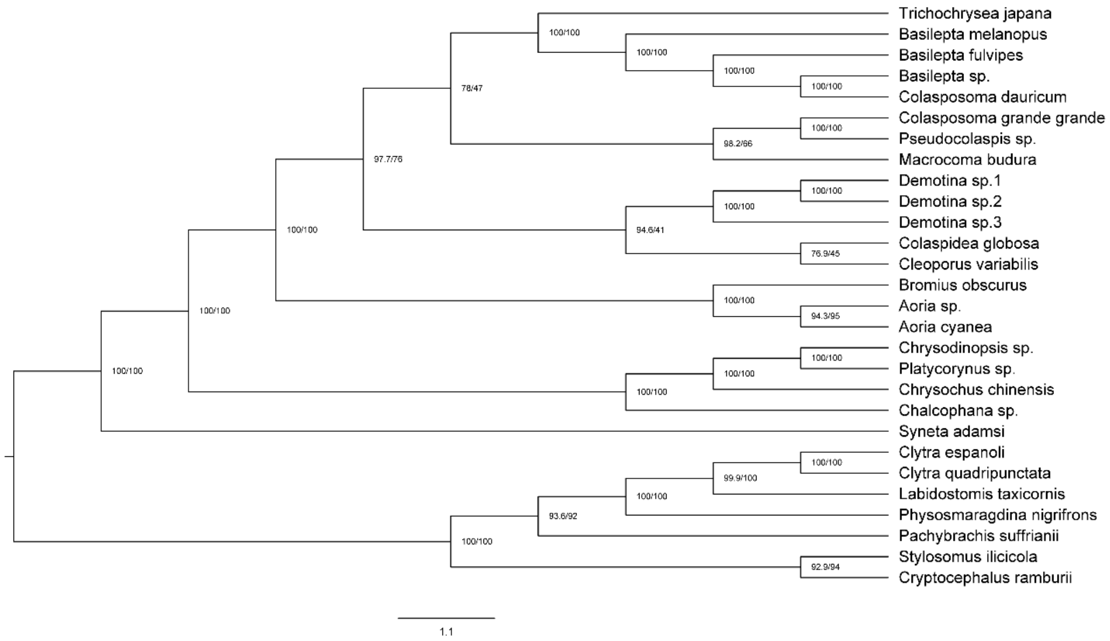

Fig. S5. Phylogenetic tree of Eumolpinae, inferred from maximum likelihood analysis (using IQ-TREE) based on 13 protein-coding genes and two ribosomal RNA genes (13PCGs + 2 rRNAs). SH-aLRT and bootstrap support values are shown near the nodes.

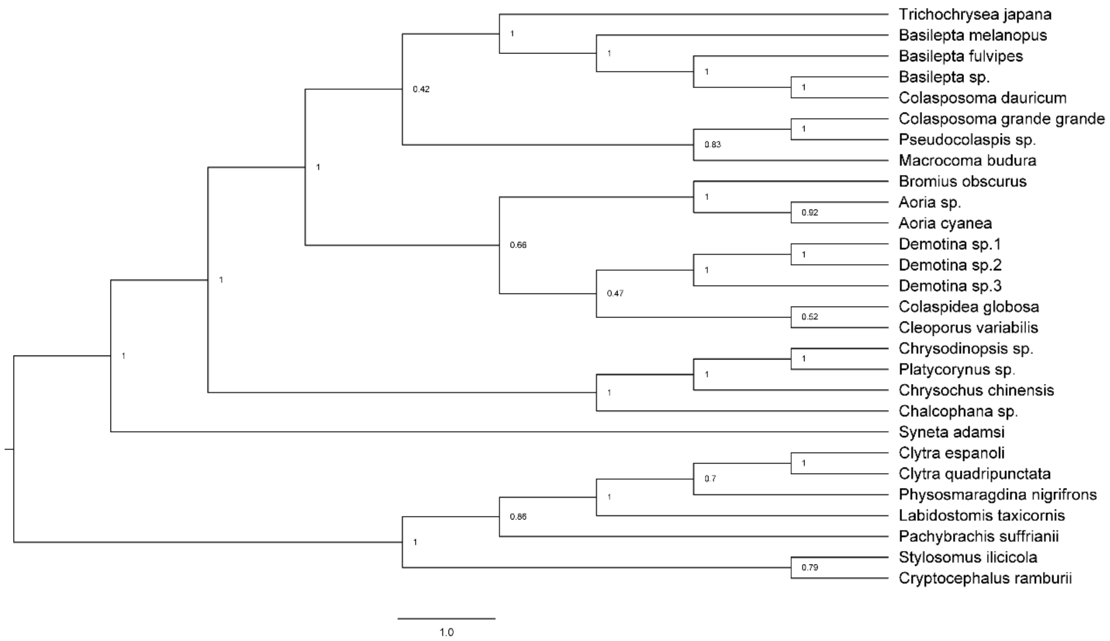

Fig. S6. Phylogenetic tree of Eumolpinae, inferred from bayesian inference (using PhyloBayes) based on 13 protein-coding genes and two ribosomal RNA genes (13PCGs + 2 rRNAs). SH-aLRT and bootstrap support values are shown near the nodes.

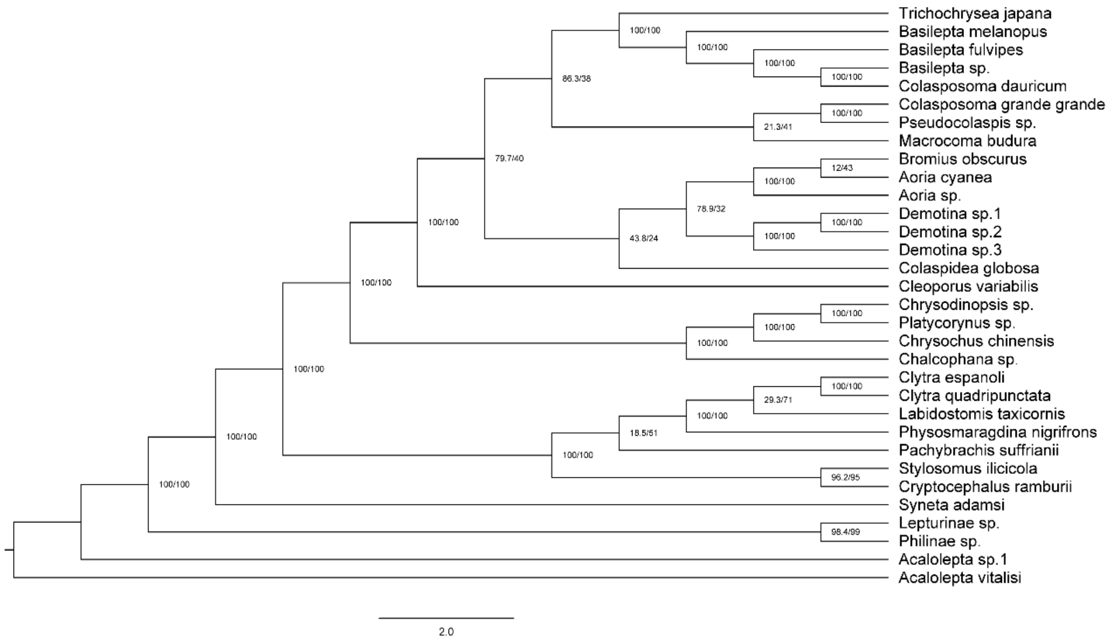

Fig. S7. Phylogenetic tree of Eumolpinae with longhorn beetles as outgroups, inferred from maximum likelihood analysis (using IQ-TREE) based on 13 PCGs were translated into amino acid (13PCGs_AA). SH-aLRT and bootstrap support values are shown near the nodes.

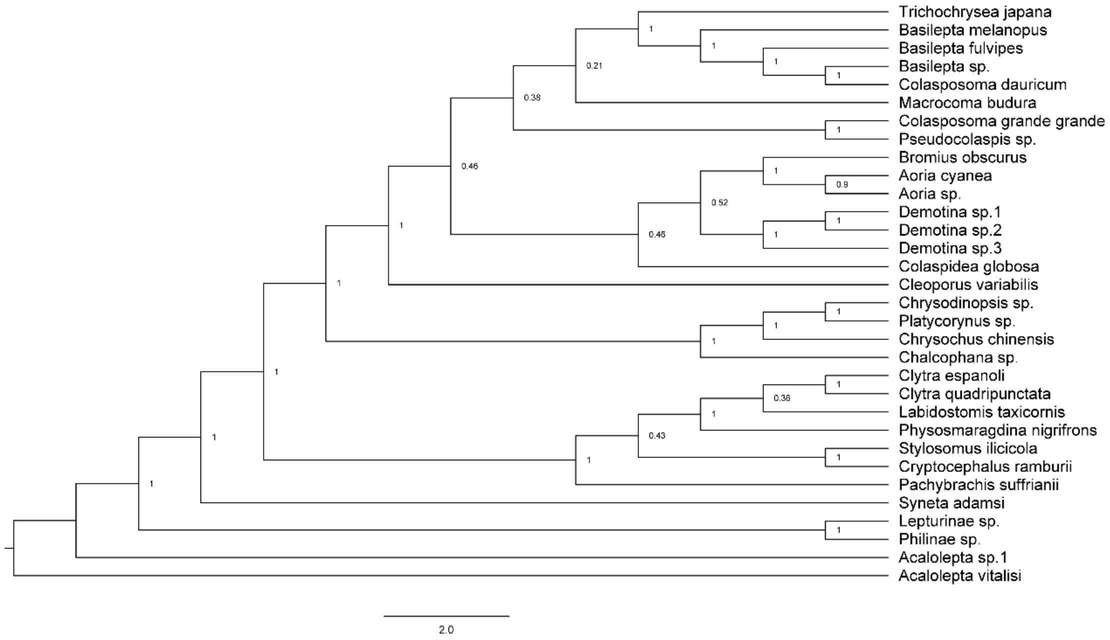

Fig. S8. Phylogenetic tree of Eumolpinae with longhorn beetles as outgroups, inferred from bayesian inference (using PhyloBayes) based on 13 PCGs were translated into amino acid (13PCGs_AA). SH-aLRT and bootstrap support values are shown near the nodes.

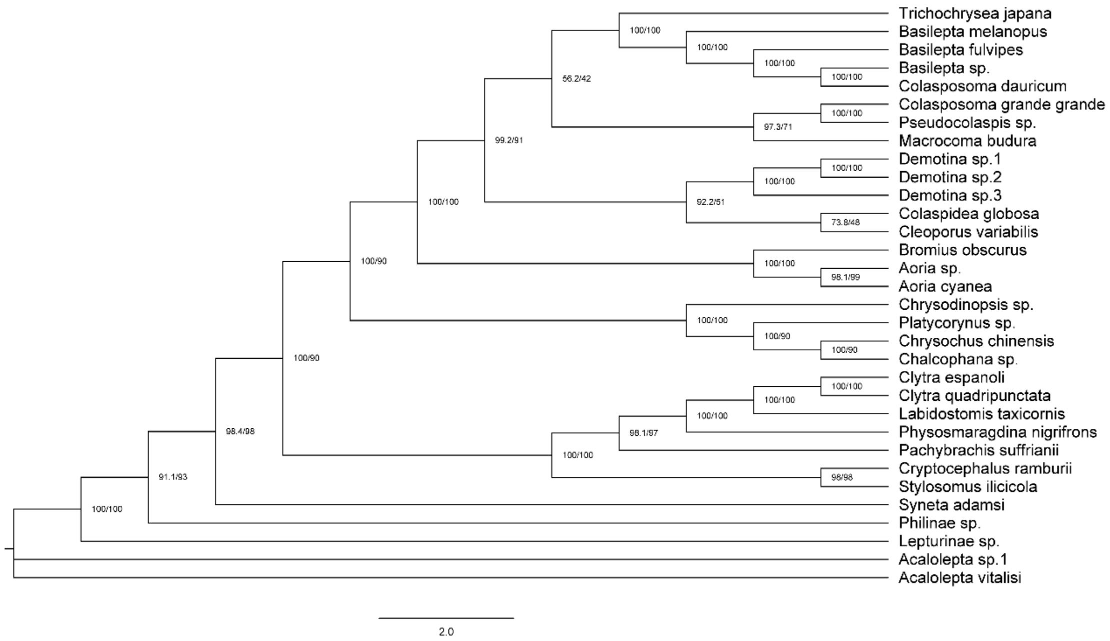

Fig. S9. Phylogenetic tree of Eumolpinae with longhorn beetles as outgroups, inferred from maximum likelihood analysis (using IQ-TREE) based on 13 protein-coding genes and two ribosomal RNA genes (13PCGs + 2 rRNAs). SH-aLRT and bootstrap support values are shown near the nodes.

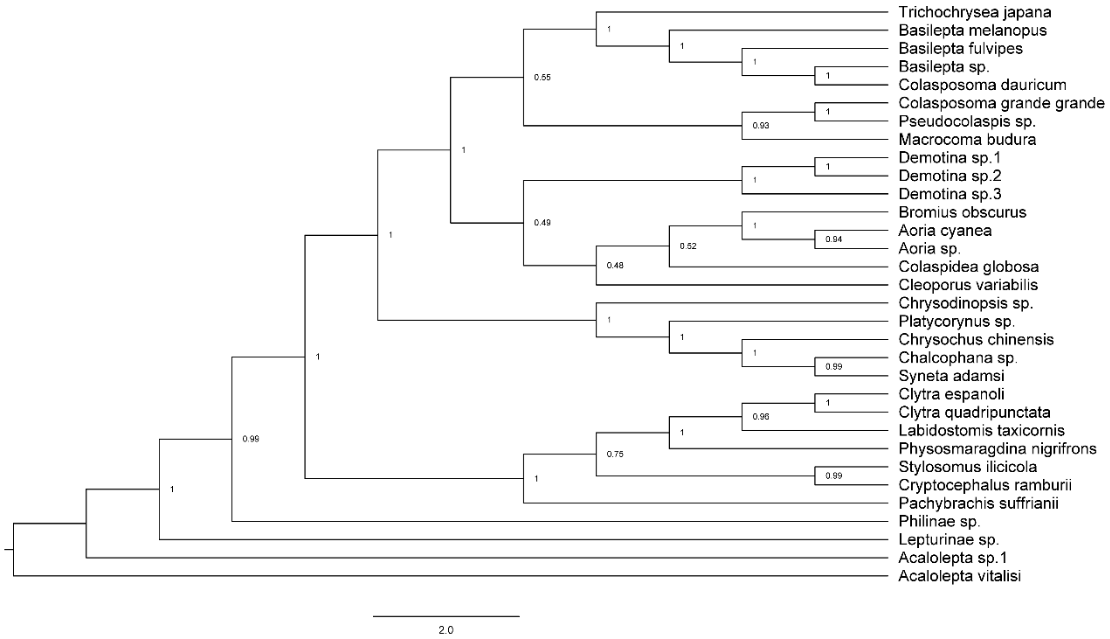

Fig. S10. Phylogenetic tree of Eumolpinae with longhorn beetles as outgroups, inferred from bayesian inference (using PhyloBayes) based on 13 protein-coding genes and two ribosomal RNA genes (13PCGs + 2 rRNAs). SH-aLRT and bootstrap support values are shown near the nodes.

Supplementary Table 1. Mitogenomic structure of the newly sequenced mitogenomes of *Macrocoma budura* and *Colasposoma grande grande* (Eumolpinae)

1. Mitogenomic structure of *Macrocoma budura*

Gene	Strand	Position（bp）	Length（bp）	Anticodon	Start codon	Stop codon	Intergenic nucleotides（bp）
trnI	J	1-63	63	GAT	-	-	0
trnQ	N	133-66	68	TTG	-	-	2
trnM	J	134-203	70	CAT	-	-	0
nad2	J	204-1217	1014	-	ATT	TAA	0
trnW	J	1216-1278	63	TCA	-	-	-2
trnC	N	1331-1272	60	GCA	-	-	-7
trnY	N	1395-1332	64	GTA	-	-	0
cox1	J	1388-2935	1548	-	ATT	TAA	-8
trnL2	J	2931-2994	64	TAA	-	-	-5
cox2	J	2995-3678	684	-	ATT	TAA	0
trnK	J	3680-3750	71	TTT	-	-	1
trnD	J	3751-3812	62	GTC	-	-	0
atp8	J	3814-3969	156	-	ATT	TAA	1
atp6	J	3963-4637	675	-	ATG	TAA	-7
cox3	J	4657-5444	788	-	ATG	T	19
trnG	J	5444-5507	64	TCC	-	-	-1
nad3	J	5508-5861	354	-	ATA	TAA	0
trnR	J	5860-5923	64	TCG	-	-	-2
trnA	J	5924-5986	63	TGC	-	-	0
trnN	J	5988-6052	65	GTT	-	-	1
trnS1	J	6052-6118	67	TCT	-	-	-1
trnE	J	6121-6183	63	TTC	-	-	2
trnF	N	6245-6182	64	GAA	-	-	-2
nad5	N	7948-6245	1704	-	ATA	TAA	-1
trnH	N	8012-7950	63	GTG	-	-	1
nad4	N	9332-8012	1321	-	ATG	T	-1
nad4L	N	9613-9326	288	-	ATG	TAG	-7
trnT	J	9617-9679	63	TGT	-	-	3
trnP	N	9743-9681	63	TGG	-	-	1
nad6	J	9745-10248	504	-	ATT	TAA	1
cytb	J	10248-11387	1140	-	ATG	TAG	-1
trnS2	J	11386-11451	66	TGA	-	-	-2
nad1	N	12420-11476	945	-	TTG	TAA	24
trnL1	N	12485-12422	64	TAG	-	-	1
rrnL	N	13754-12486	1269	-	-	-	0
trnV	N	13820-13757	64	TAC	-	-	2

rrnS	N	14567-13824	744	-	-	-	3
AT-rich region	J	14568-15348	781	-	-	-	0

2. Mitogenomic structure of *Colasposoma grande grande*

Gene	Strand	Position（bp）	Length（bp）	Anticodon	Start codon	Stop codon	Intergenic nucleotides（bp）
trnI	J	1-63	63	GAT	-	-	0
trnQ	N	136-68	69	TTG	-	-	4
trnM	J	136-205	70	CAT	-	-	-1
nad2	J	206-1213	1008	-	ATA	TAA	0
trnW	J	1212-1277	66	TCA	-	-	-2
trnC	N	1331-1271	61	GCA	-	-	-7
trnY	N	1396-1332	65	GTA	-	-	0
cox1	J	1389-2936	1548	-	ATT	TAA	-8
trnL2	J	2932-2995	64	TAA	-	-	-5
cox2	J	2996-3679	684	-	ATC	TAA	0
trnK	J	3681-3751	71	TTT	-	-	1
trnD	J	3752-3817	66	GTC	-	-	0
atp8	J	3818-3973	156	-	ATA	TAA	0
atp6	J	3967-4641	675	-	ATG	TAA	-7
cox3	J	4641-5427	787	-	ATG	T	-1
trnG	J	5428-5491	64	TCC	-	-	0
nad3	J	5492-5845	354	-	ATC	TAA	0
trnR	J	5844-5906	63	TCG	-	-	-2
trnA	J	5907-5971	65	TGC	-	-	0
trnN	J	5972-6035	64	GTT	-	-	0
trnS1	J	6036-6102	67	TCT	-	-	0
trnE	J	6103-6165	63	TTC	-	-	0
trnF	N	6229-6164	66	GAA	-	-	-2
nad5	N	7934-6230	1705	-	ATA	T	0
trnH	N	7998-7935	64	GTG	-	-	0
nad4	N	9316-7999	1318	-	ATA	T	0
nad4L	N	9600-9313	288	-	ATG	TAG	-4
trnT	J	9605-9668	64	TGT	-	-	4
trnP	N	9731-9669	63	TGG	-	-	0
nad6	J	9734-10216	483	-	ATT	TAA	2
cytb	J	10216-11355	1140	-	ATG	TAG	-1
trnS2	J	11354-11420	67	TGA	-	-	-2
nad1	N	12384-11443	942	-	TTG	TAA	22
trnL1	N	12447-12386	62	TAG	-	-	1
rrnL	N	13696-12448	1249	-	-	-	0
trnV	N	13794-13731	64	TAC	-	-	34

rrnS	N	14541-13794	748	-	-	-	-1
AT-rich region	J	14542-15558	1017	-	-	-	0

Note: Direction **J** indicates the majority (or main) strand, while **N** indicates the minority (or secondary) strand. A **negative number** represents gene overlap, a **positive number** indicates intergenic spacing, and **0** means there is no gap or overlap between the genes.

Note: tRNA loci are given using the compact trnX notation (e.g., *trnL1* = tRNA-Leu [UUR], *trnL2* = tRNA-Leu [CUN], *trnS1* = tRNA-Ser [AGN], *trnS2* = tRNA-Ser [UCN]). *rrnL* = 16S rRNA, *rrnS* = 12S rRNA. The AT-rich region is listed as control region.

Supplementary Table 2. Base composition of the mitogenomes of *Macrocoma budura* and *Colasposoma grande grande*

1. Nucleotide composition of the mitochondrial genome of *Macrocoma budura*

Region / Gene	Size (bp)	A (%)	C (%)	G (%)	T (%)	A+T (%)	G+C (%)	AT-skew	GC-skew
**Whole genome**	15,374	41.9	14.1	8.7	35.4	77.3	22.8	0.084	−0.237
**Protein-coding genes**	9,570	33.1	11.1	11.5	44.3	77.4	22.6	−0.145	0.018
**1st codon position**	3,190	34.8	11.2	16.3	37.7	72.5	27.5	−0.040	0.185
**2nd codon position**	3,190	21.1	16.4	13.4	49.2	70.3	29.8	−0.400	−0.101
**3rd codon position**	3,190	43.5	5.8	4.8	45.9	89.4	10.6	−0.027	−0.094
**rrnS**	744	44.2	13.2	6.6	36.0	80.2	19.8	0.102	−0.333
**rrnL**	1,269	45.4	12.8	6.4	35.4	80.8	19.2	0.124	−0.333
**nad1**	945	27.9	7.4	15.3	49.3	77.2	22.7	−0.277	0.348
**cytb**	1,140	35.8	15.2	10.8	38.2	74.0	26.0	−0.032	−0.169
**nad6**	504	44.4	11.9	5.2	38.5	82.9	17.1	0.071	−0.392
**nad4L**	288	28.1	3.8	12.8	55.2	83.3	16.6	−0.325	0.542
**nad4**	1,321	27.0	7.2	14.5	51.2	78.2	21.7	−0.309	0.336
**nad5**	1,704	30.3	7.2	13.0	49.5	79.8	20.2	−0.241	0.287
**cox1**	1,543	33.2	16.3	14.1	36.4	69.6	30.4	−0.046	−0.072
**cox2**	684	36.7	16.2	10.8	36.3	73.0	27.0	0.005	−0.200
**atp8**	156	42.3	13.5	4.5	39.7	82.0	18.0	0.032	−0.500
**atp6**	675	36.3	15.0	8.3	40.4	76.7	23.3	−0.053	−0.288
**cox3**	788	32.7	14.8	12.9	39.5	72.2	27.7	−0.094	−0.069
**nad3**	354	35.9	12.1	9.0	42.9	78.8	21.1	−0.089	−0.147
**nad2**	1,014	37.0	13.8	8.1	41.1	78.1	21.9	−0.052	−0.260
**Control region**	781	41.2	14.9	6.5	37.4	78.6	21.4	0.048	−0.393

2. Nucleotide composition of the mitochondrial genome of *Colasposoma grande grande*

Region / Gene	Size (bp)	A (%)	C (%)	G (%)	T (%)	A+T (%)	G+C (%)	AT-skew	GC-skew
**Whole genome**	15,565	42.5	14.9	8.7	33.9	76.4	23.6	0.113	−0.263
**Protein-coding genes**	11,085	32.8	12.8	11.9	42.5	75.3	24.7	−0.129	−0.036
**1st codon position**	3,695	34.8	11.8	17.4	35.9	70.7	29.2	−0.016	0.192
**2nd codon position**	3,695	20.7	18.5	13.6	47.3	68.0	32.1	−0.391	−0.153
**3rd codon position**	3,695	42.9	8.2	4.7	44.2	87.1	12.9	−0.015	−0.271
**rrnS**	748	34.5	7.1	15.0	43.4	77.9	22.1	−0.114	0.357
**rrnL**	1,249	34.2	6.6	14.2	45.0	79.2	20.8	−0.136	0.365
**nad1**	942	26.4	8.4	15.4	49.8	76.2	23.8	−0.307	0.294
**cytb**	1,140	34.2	18.2	10.7	36.9	71.1	28.9	−0.038	−0.260
**nad6**	483	43.7	13.9	5.2	37.3	81.0	19.1	0.079	−0.455
**nad4L**	288	26.4	4.9	13.9	54.9	81.3	18.8	−0.351	0.479
**nad4**	1,318	27.3	7.1	13.9	51.7	79.0	21.0	−0.309	0.324
**nad5**	1,705	28.2	7.5	13.5	50.8	79.0	21.0	−0.286	0.286
**cox1**	1,548	32.7	17.6	13.9	35.8	68.5	31.5	−0.045	−0.117
**cox2**	684	36.8	17.0	11.4	34.8	71.6	28.4	0.028	−0.197

**atp8**	156	46.8	14.1	4.5	34.6	81.4	18.6	0.150	−0.516
**atp6**	675	36.7	14.7	9.0	39.6	76.3	23.7	−0.038	−0.241
**cox3**	787	33.9	15.9	13.6	36.6	70.5	29.5	−0.038	−0.078
**nad3**	354	35.3	15.5	9.6	39.5	74.8	25.1	−0.056	−0.235
**nad2**	1,008	39.7	14.3	7.1	38.9	78.6	21.4	0.010	−0.336
**Control region**	1,017	45.3	13.0	4.6	37.1	82.4	17.6	0.100	−0.477
